# Comparison of postoperative infection risk with and without topical vancomycin in adult posterior spinal fusion: a propensity-matched cohort study of 126,910 patients

**DOI:** 10.1007/s43390-026-01378-y

**Published:** 2026-04-27

**Authors:** Haad A. Arif, Devan Devkumar, Hesham Tanbour, Michael J. Conklin, Steven M. Theiss

**Affiliations:** Department of Orthopaedic Surgery, University of Alabama at Birmingham, Birmingham, Alabama USA

**Keywords:** Spine, Fusion, Vancomycin, Gentamicin, Tobramycin, Surgical site infection, Posterior approach, Topical prophylaxis, Antibiotic

## Abstract

**Purpose:**

Surgical site infections (SSIs) are the third most common complication following spine surgery. The effect of topical vancomycin in addition to perioperative systemic cefazolin remains uncertain. This study sought to investigate the effect of topical vancomycin administration on 90-day postoperative infection risk following adult posterior spinal fusion (PSF).

**Methods:**

The TriNetX Global Collaborative database was queried to identify adult patients undergoing PSF. Patients were stratified into two cohorts based on documented administration of topical vancomycin and systemic cefazolin (topical cohort) versus systemic cefazolin alone (control cohort). Patients underwent 1:1 propensity matching based on demographics and relevant comorbidities. The primary outcomes of interest included 90-day rates of infection-related complications. Subgroup analyses were performed to identify the influence of neuromuscular scoliosis, PSF with osteotomies, pelvic fixation, or fusion of greater than six levels on infection rates. Infectious complication rates were also compared between patients receiving topical vancomycin with and without topical gentamicin or tobramycin.

**Results:**

After propensity matching, 63,455 patients were included in each cohort. Patients in the topical cohort demonstrated comparable rates of overall infection (3.0% vs. 3.1%, *p* = 0.794), superficial surgical site infection (SSI) (0.9% vs. 0.8%, *p* = 0.151), deep SSI (0.6% vs. 0.6%, *p* = 0.070) and all other infectious complications when compared to the control cohort. Subgroup analysis revealed decreased rate of deep SSI when using topical gentamicin or tobramycin with vancomycin versus topical vancomycin alone (0.7% vs. 1.7%, *p* = 0.019). No other between-group differences were observed.

**Conclusion:**

This study found no significant difference in 90-day postoperative infectious complications following PSF when topical vancomycin was added to standard systemic cefazolin prophylaxis compared to cefazolin prophylaxis alone. Vancomycin with the addition of gentamicin or tobramycin, however, was associated with decreased rates of deep SSI.

**Level of evidence:**

Level III, retrospective cohort study.

**Supplementary Information:**

The online version contains supplementary material available at https://doi.org/10.1007/s43390-026-01378-y.

## Introduction

Surgical site infections (SSIs) contribute to an estimated 8000 deaths annually and healthcare expenditures reaching 10 billion USD [1]. These complications can be defined as superficial, deep, or organ space, with treatment options ranging from medical antibiotic management for superficial infections to operative irrigation and debridement for deep and organ space infections [2, 3]. Representing the third most common complication following spinal surgery, SSIs are associated with increased postoperative complications such as pseudoarthrosis and neurologic insult, increased healthcare utilization and subsequent costs, and greater patient morbidity [2–4]. Several risk factors for SSI following spine surgery have been identified including advanced age, diabetes, obesity, hypertension, blood transfusion, and longer operative times [3, 5]. Additionally, surgery for neuromuscular scoliosis (NMS), posterior surgical approaches, and instrumented procedures all increase the risk of postoperative SSI. [3, 6].

Prevention of SSI in spine surgery rests upon perioperative prophylactic antibiotics, typically systemic, intravenous (IV) cefazolin due to its broad spectrum antimicrobial profile [2, 5, 7]. However, increased use of cephalosporins leading to microbial resistance and persistent rates of SSI have prompted additional efforts in SSI prevention [2, 5]. One strategy is the use of topical vancomycin administered locally in the operative site prior to wound closure [8, 9]. Despite several side effects when delivered systemically, including hypotension, renal toxicity, and colitis, topical vancomycin offers a promising adjunct in SSI prevention due to its affordability, high local concentration, limited systemic toxicity, and weak inhibition of osteogenic activity [10, 11].

Still, the use of topical vancomycin in spine surgery remains controversial. Many studies report conflicting findings regarding the efficacy of topical vancomycin and are limited by small sample size, heterogenous study populations, and differences in study design [2, 4, 5, 12–16]. Moreover, vancomycin has not yet been approved for local administration by the Food and Drug Administration [13]. While the North American Spine Society clinical guidelines suggest the use of topical vancomycin in select patient populations, a recent online survey among international AO spine members reflects ongoing debate regarding its utility in adult spinal correction [8, 17].

We sought to compare the rate of postoperative infectious complications, including overall infection and SSI, in adult patients undergoing posterior spinal fusion (PSF) with both topical vancomycin and perioperative systemic cefazolin to those receiving systemic cefazolin alone. Our secondary aims were to compare the rates of postoperative acute kidney injury (AKI) and the efficacy of concurrent topical vancomycin with gentamicin or tobramycin. We hypothesized that administration of local vancomycin lowers the risk of infectious complications without an associated increased risk of postoperative AKI.

## Materials and methods

### Data source

This retrospective cohort study utilized the TriNetX Global Collaborative Network, a database platform composed of electronic medical records of over 196 million patients from 163 institutions across 30 countries. TriNetX has been used extensively in orthopedic research and provides access to de-identified and anonymized patient data [18–25]. The database extracts both structured and unstructured data from discrete EMR fields and clinical notes and converts them into retrievable Current Procedural Terminology (CPT) codes and International Classification of Diseases, 10th Revision (ICD10) codes. Medication data, including inpatient and outpatient prescriptions, are captured through Veterans Affairs (VA) RxNorm codes. To ensure accuracy, all data are reviewed and vetted prior to uploading to the TriNetX platform. This study did not require Institutional Review Board approval as all work was a secondary analysis of existing, de-identified data. This study adhered to the Strengthening the Reporting of OBservational studies in Epidemiology (STROBE) guidelines for all data reporting.

### Cohort design

Methodology for cohort design mirrored that as described by Chopra et al. [21]. Using ICD10 and CPT codes, all adult patients over the age of 18 years undergoing posterior spinal instrumentation (CPT 22842, 22,843, or 22,844) and fusion (CPT 22800, 22,802, or 22,804) between 2011 and 2024 were identified. The year 2011 was selected as the beginning of the study period to coincide with early reports of topical vancomycin use in spine surgery [26] and ensure contemporaneous surgical practice patterns. Patients were then separated into two cohorts. The topical cohort was defined as patients receiving both systemic perioperative cefazolin and topical vancomycin on the day of the index PSF operation (within 24 hours from surgery). Administration routes were specified through built-in filters available on the TriNetX platform drawn from both medication orders and narrative clinical notes documented within patients’ electronic medical records. This approach allows differentiation of topical from systemic administration but remains dependent on institutional documentation practices. The control cohort was defined as patients receiving only systemic perioperative cefazolin within 24 hours of surgery without any topical vancomycin administration. Exclusion criteria were defined as patients with a history of osteomyelitis within six months of PSF (M46.2), history of previous PSF within 10 years, and patients with documented insertion of a drug delivery device (CPT 11981 or 20,700) on the day of PSF [2, 21].

### Propensity score matching

Given the documented risk factors and potential for confounding variables, propensity score matching was employed to ensure a balanced comparison. Matching was done using a 1:1 ratio and a greedy nearest-neighbor logistic regression algorithm with a caliper of 0.1 standard pooled deviations. Balanced cohorts were verified with a standard mean difference less than 0.1. Covariates including patient age and sex, diabetes mellitus, hypertension, obesity, ischemic heart disease, smoking status (present/absent) or tobacco use (F17.21, F17.22, F17.29, or Z72.0), cancer, chronic kidney disease, and personal history of methicillin-resistant Staphylococcus aureus (MRSA) infection were selected due to their influence on the outcome measures and documented use in the literature [21, 22, 27].

### Outcome measures

The primary outcome measure of interest was the difference in rate of overall infection (ICD10 T81.4) and superficial and deep SSI (ICD10 T81.41 and T81.42, respectively) between the topical and control cohorts at 90 days postoperatively. Secondary outcomes included 90-day rates of the following: postoperative sepsis (ICD10 T81.44), wound disruption (ICD10 T81.3), bacteremia (ICD10 R78.81), reoperation for exploration or irrigation and debridement (CPT 22010, 22,015, 22,830, or 10,180), and AKI (ICD10 N17).

Subgroup analyses were conducted to further investigate factors influencing the risk of infection following PSF. These include patients undergoing PSF for neuromuscular scoliosis (ICD10 M41.1), fusion of more than six levels as a proxy for operative duration, the presence of posterior or posterolateral spinal osteotomies (CPT 22206—22,208, 22,210, 22,212, 22,214, or 22,216) as a proxy for operative complexity, and PSF with pelvic fixation (CPT 22848) [3, 6]. These factors were selected based on prior literature demonstrating their association with higher postoperative SSI risk in spine surgery [28–31]. An additional subgroup analysis within the topical vancomycin cohort was performed to assess the utility of combined topical vancomycin and topical gentamicin or tobramycin for added gram-negative coverage. Secondary, infectious-related outcomes were assessed including systemic gram-positive infections (ICD10 A40, A41.0—A41.2, or B95) and systemic gram-negative infections (ICD10 A41.5, B96.1, B96.2, B96.4, or B96.5). Notably, because ICD10 organism attribution codes for gram-positive/negative coverage primarily indicate causative organisms rather than discrete clinical events, these outcomes were interpreted descriptively and were not considered primary endpoints. Patients lacking data for a given outcome were excluded only from analyses of that specific outcome and remained included in all other outcome analyses.

### Statistical analysis

Statistical analyses were performed using the built-in analytic tools available on the TriNetX platform, which provide standardized cohort comparison methods but do not allow user-specified regression or generalized linear modeling. Statistical calculations were performed using Java, R, and Python scripts. Baseline demographics were presented as a mean ± standard deviation (SD) for continuous variables and as proportions (n [%]) for categorical variables. P-values and risk ratios (RR) were used to compare differences between cohorts with the statistical significance threshold set at *p* < 0.05 a priori.

## Results

**Fig. 1:**
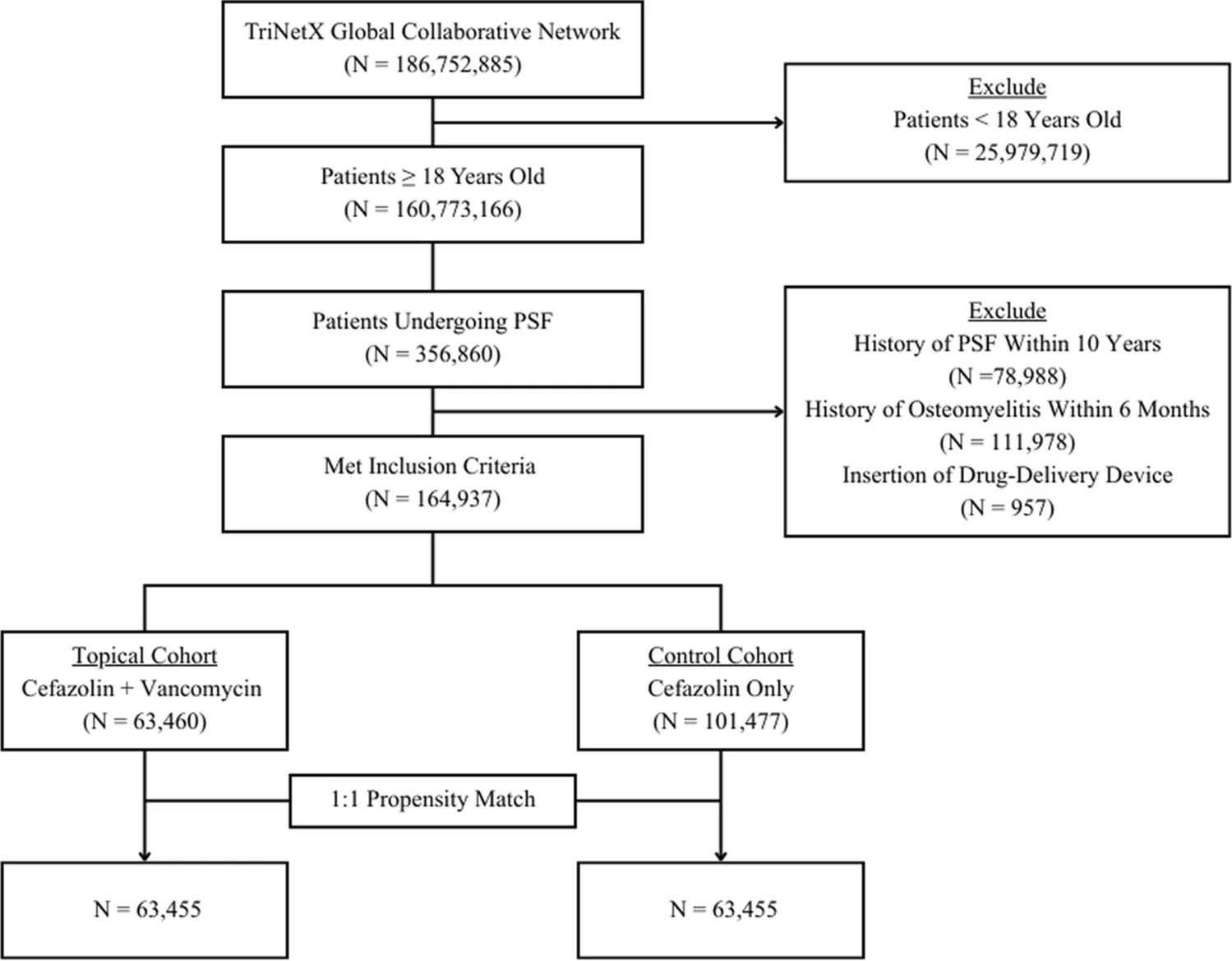
Cohort selection process

A total of 164,937 adult patients undergoing PSF were identified in this study. Topical vancomycin and systemic cefazolin were used in 63,460 (38.5%) patients while systemic cefazolin alone was used in 101,477 (61.5%) patients. Figure 1 demonstrates the cohort selection process. Baseline characteristics were assessed before and after propensity score matching (Supplemental Digital Content 1). After propensity score matching, both cohorts consisted of 63,455 patients for a total sample size of 126,910 patients. No significant differences were identified in baseline characteristics between both cohorts, ensuring adequate matching of the 126,910 patients used in the following assessments.

The overall infection rate at 90 days was 3.1% with no difference observed between the topical and control cohorts (3.0% vs. 3.1%, *p* = 0.794, RR: 0.99). Patients in both cohorts had comparable rates of superficial and deep SSI, postoperative sepsis, wound disruption, bacteremia, reoperation, and AKI (Table 1).

**Table 1:** Overall comparison of 90-day postoperative complication rates in patients with and without topical vancomycin

Outcome	Topical *n* (%)	Control *n* (%)	*P* Value	Risk ratio
Overall infection	1925 (3.0)	1941 (3.1)	0.792	0.99
Superficial SSI	540 (0.9)	494 (0.8)	0.151	1.09
Deep SSI	408 (0.6)	358 (0.6)	0.070	1.14
Wound disruption	2142 (3.4)	2018 (3.2)	0.051	1.06
Postoperative sepsis	75 (0.1)	71 (0.1)	0.741	1.06
Bacteremia	1242 (2.0)	1214 (1.9)	0.568	1.02
Reoperation	1272 (2.0)	1267 (2.0)	0.920	1.00
Acute kidney injury	3057 (4.8)	3003 (4.7)	0.477	1.02

*n* = *63,455; SSI Surgical site infection*

### Topical vancomycin with and without gram-negative coverage

A total of 64,966 PSF patients received systemic cefazolin and topical vancomycin. Of these, 1506 (2.3%) patients also received gram-negative coverage with topical gentamicin or tobramycin and were matched to the remaining 63,460 (97.7%) patients without any topical gram-negative coverage yielding a total cohort of 3012 patients (1506 patients per group). Patients receiving topical vancomycin and gram-negative coverage demonstrated decreased rates of deep SSI (0.7% vs. 1.7%, *p* = 0.019, RR: 0.44) compared to patients receiving topical vancomycin alone. Patients with topical gram-negative coverage also demonstrated decreased rates of reoperation (1.3% vs. 2.1%, *p* = 0.093, RR: 0.63), although this did not reach statistical significance. Both cohorts had comparable rates of overall infection (3.3% vs. 4.3%, *p* = 0.127, RR: 0.75), superficial SSI (1.1% vs. 1.2%, *p* = 0.7301, RR: 0.889), wound disruption (3.9% vs. 4.3%, *p* = 0.528, RR: 0.91), systemic gram-positive infection (2.3% vs. 1.7%, p = 0.244, RR: 1.35), and systemic gram-negative infection (3.3% vs. 3.5%, *p* = 0.840, RR: 0.96). Rates of postoperative sepsis could not be evaluated due to TriNetX privacy policies which obfuscate outcome comparisons with patient counts below 10.

### Pelvic fixation

A total of 14,571 patients underwent PSF with pelvic fixation. Of these, 6392 (43.9%) patients received topical vancomycin and were matched to 8179 (56.1%) control patients receiving only systemic cefazolin, yielding a total cohort of 12,784 patients (6392 patients per group). Subgroup analysis revealed patients in the topical vancomycin cohort to demonstrate comparable rates of all measured outcomes to the control cohort when fusion extended to the pelvis (Table 2).

**Table 2:** Comparison of 90-day postoperative complication rates in psf including pelvic fixation

Outcome	Topical *n* (%)	Control *n* (%)	*P* Value	Risk ratio
Overall infection	281 (4.4)	286 (4.5)	0.830	0.98
Superficial SSI	77 (1.2)	76 (1.2)	0.935	1.01
Deep SSI	67 (1.0)	73 (1.1)	0.610	0.92
Wound disruption	339 (5.3)	337 (5.3)	0.937	1.01
Postoperative sepsis	14 (0.2)	11 (0.2)	0.548	1.27
Bacteremia	133 (2.1)	136 (2.1)	0.853	0.98
Reoperation	196 (3.1)	202 (3.2)	0.760	0.97
Acute kidney injury	400 (6.3)	387 (6.1)	0.632	1.03

*N* = *6392 patients per cohort*

### Neuromuscular scoliosis

A total of 2430 patients with NMS underwent PSF. Of these, 893 (36.7%) patients received topical vancomycin and were matched to 1537 (63.3%) control patients receiving only systemic cefazolin, yielding a total cohort of 1786 patients (893 patients per group). The overall infection rate across both cohorts was 4.5%. Patients in the topical cohort demonstrated comparable rates of overall infection (5.2% vs. 3.9, *p* = 0.211, RR: 1.31), wound disruption (7.4% vs. 6.0%, *p* = 0.257, RR: 1.22), bacteremia (2.6% each, *p* = 1.000, RR: 1.00), reoperation (4.9% vs. 4.0%, *p* = 0.360, RR: 1.22), and AKI (1.5% vs. 1.7%, *p* = 0.703, RR: 0.87) when compared to the control cohort. Rates of superficial and deep SSI, and postoperative sepsis could not be assessed due patient counts below 10.

### Fusion construct greater than six levels

A total of 13,331 patients underwent PSF of greater than six levels. Of these, 4854 (36.4%) patients received topical vancomycin and were matched to 8477 (63.6%) control patients receiving only systemic cefazolin, yielding a total cohort of 9688 patients (4854 patients per group). No between-group differences were observed in any of the outcome measures, though postoperative sepsis could not be evaluated due to patient counts below 10 (Table 3).

**Table 3:** Comparison of 90-day postoperative complication rates in psf of greater than six levels

Outcome	Topical *n* (%)	Control *n* (%)	*P* Value	Risk ratio
Overall infection	121 (2.5)	121 (2.5)	1.000	1.00
Superficial SSI	29 (0.6)	31 (0.6)	0.796	0.94
Deep SSI	26 (0.5)	24 (0.5)	0.777	1.08
Wound disruption	152 (3.1)	133 (2.7)	0.253	1.14
Postoperative sepsis	-	-	-	-
Bacteremia	60 (1.2)	58 (1.2)	0.853	1.03
Reoperation	88 (1.8)	86 (1.8)	0.878	1.02
Acute kidney injury	94 (1.9)	94 (1.9)	1.000	1.00

*N* = *4854 patients per cohort*

### Spinal osteotomy

A total of 7129 patients underwent PSF with additional posterior/posterolateral osteotomies. Of these, 2864 (40.2%) patients received topical vancomycin and were matched to 4265 (59.8%) control patients receiving only systemic cefazolin yielding a total cohort of 5728 patients (2864 patients per group). No between-group differences were observed in any of the outcome measures, though postoperative sepsis could not be evaluated due to patient counts below 10 (Table 4).

**Table 4:** Comparison of 90-day postoperative complication rates in psf with osteotomies

Outcome	Topical *n* (%)	Control *n* (%)	*P* Value	Risk ratio
Overall infection	96 (3.4)	101 (3.5)	0.717	0.95
Superficial SSI	28 (1.0)	27 (0.9)	0.892	1.04
Deep SSI	25 (0.9)	26 (0.9)	0.888	0.96
Wound disruption	101 (3.5)	99 (3.5)	0.886	1.02
Postoperative sepsis	-	-	-	-
Bacteremia	47 (1.6)	49 (1.7)	0.837	0.96
Reoperation	76 (2.7)	76 (2.7)	1.000	1.00
Acute kidney injury	93 (3.2)	91 (3.2)	0.881	1.02

*N* = *2864 patients per cohort*

## Discussion

In this large, propensity-matched cohort of over 126,000 adult patients undergoing instrumented PSF, the addition of topical vancomycin to systemic perioperative cefazolin was not associated with a significant reduction in infectious complications at 90 days postoperatively. Patients receiving concurrent topical gentamicin or tobramycin, however, demonstrated a lower incidence of deep SSI, suggesting a possible synergistic benefit of broader antimicrobial coverage. Infectious complication rates were otherwise comparable across all other subgroup analyses, including patients undergoing pelvic fixation, long fusion constructs, spinal osteotomies, and patients with NMS.

These findings align with recent large-scale analyses reporting limited benefit of topical vancomycin in spine surgery in the setting of systemic perioperative cefazolin use [4, 12, 13, 16]. However, this contrasts with several earlier single-center studies and meta-analyses that reported significant reductions in deep SSI rates with topical vancomycin use [2, 11, 14, 22, 32]. Additional studies investigating specific patient populations such as spinal tumors or fractures have also demonstrated decreased SSI incidence with topical vancomycin use [33–35]. The discrepancy between these prior reports and the current study may reflect differences in study design, patient selection, and exposure definition [35, 36]. For instance, although Karnati et al. cite a significant reduction in postoperative infection following lumbar spine surgery, the use of topical vancomycin was inferred based on the surgical date (before vs. after December 31, 2013) rather than documented administration [22]. In contrast, the present study employs TriNetX medication data and route of administration to specify use of intravenous cefazolin and topical vancomycin, gentamicin, and tobramycin. Furthermore, the data reported here encompass a wide range of spinal fusion procedures and indications rather than focusing on a limited subset of operations. As such, the findings discussed in this study must be considered in context with overall limitations inherent to database analyses.

It is also possible that, although vancomycin extends antimicrobial coverage to include MRSA, its limited gram-negative and anaerobic coverage may explain its inconsistent clinical efficacy in prior works [15, 37]. Accordingly, the lack of a measurable reduction in SSI rates in the topical cohort suggests that standard intravenous cefazolin provides adequate prophylaxis against the most common pathogens in instrumented PSF [15]. However, a nearly 60% reduction in deep SSI incidence was observed when topical gentamicin or tobramycin was added to vancomycin (0.7% vs. 1.7%, *p* = 0.019, RR: 0.44). Although the absolute differences in infection rates were small, these differences may still represent considerable implications when extrapolated to the population level given the morbidity, cost, and readmission burden associated with postoperative spinal infections. Prior cost analyses have demonstrated considerable cost-saving effect of topical vancomycin, saving nearly $250,000 per 100 complex spinal procedures and reducing return-to-surgery rates [32, 38, 39].

Although gram-positive organisms such as Staphylococcus aureus and coagulase-negative Staphylococcus species account for the majority of spinal surgical site infections, prior studies have reported that gram-negative and polymicrobial infections represent up to 50% of surgical site infections following lumbar spine surgery [40–43]. In this context, the addition of aminoglycosides may provide broader antimicrobial coverage while also exhibiting synergistic activity with vancomycin against certain gram-positive organisms [44–46]. Moreover, aminoglycosides have demonstrated activity against Staphylococcus biofilm formation at bactericidal concentrations, which may further contribute to infection prevention in the setting of implanted instrumentation [47]. Because microbiologic culture data were not available within the TriNetX platform, the pathogen distribution in the present cohort could not be characterized. Accordingly, this finding should be interpreted as exploratory and hypothesis-generating and warrants further investigation in prospective studies with detailed microbiologic data and in higher-risk settings.

The current study also found no increased risk of AKI in patients receiving topical vancomycin, reinforcing its safety profile and suggesting minimal systemic absorption [48, 49]. Additionally, the use of topical vancomycin has been shown to have minimal impact on antibiotic resistance [7]. Collectively, the results of this study suggest that while routine use of topical vancomycin in PSF may not significantly reduce postoperative infection risk, its combination with aminoglycosides such as gentamicin or tobramycin, may be considered in high-risk settings without added systemic risk.

### Limitations

This study has several limitations that warrant consideration. The retrospective design precludes causal inference, and reliance on ICD-10 and CPT codes introduces potential for classification bias. Furthermore, aggregating all PSF indications limits evaluation of procedure-specific infection risks. Operative variables such as antibiotic dosing, delivery method (powder vs. beads vs. mixing with bone graft), blood loss, adjunct antimicrobial strategies (surgical drains or povidone-iodine irrigation) [50] and surgical duration were unavailable. In addition, TriNetX does not consistently capture exact topical antibiotic dose, preparation, and timing relative to wound closure, limiting standardization of exposure across institutions. It is possible that significant differences between various methods of local antibiotic delivery exist and were not captured in this study. As such, the null result in infection difference between the topical and control cohorts in this study must be interpreted cautiously. Because TriNetX does not report on detailed microbiologic culture data, timing of infection onset, or operative findings at reoperation, characterization of infection phenotypes and evaluation of pathogen-specific risk, shifts in bacterial flora, or treatment patterns were not available for evaluation. Additionally, concomitant medication or antibiotic use other than vancomycin cefazolin or gentamycin/tobramycin was not captured in the analysis, increasing the risk for heterogeneity in the analyzed cohorts. Such heterogeneity may introduce exposure contamination across cohorts. Although the present study is sufficiently powered to detect moderate effect sizes, the relatively low overall infection rate may limit its ability to identify smaller but clinically meaningful differences. Future prospective, randomized studies with stratified enrollment by risk profile could better determine whether vancomycin confers incremental benefit in specific subgroups. There is also a risk of misclassification inherent to analyses using a multinational network such as TriNetX. Participating institutions may differ in both clinical practice patterns and documentation standards, which could affect the accuracy and consistency of exposure and outcome capture across sites. Furthermore, residual confounding remains an important limitation. Although propensity score matching was employed, critical operative and perioperative variables such as revision status, use of surgical drains, wound classification, intraoperative antiseptic irrigation practices, closure techniques, and institution-specific infection prevention protocols could not be incorporated into the matching algorithm and may have influenced infection risk. The absence of adjustment for multiple comparisons increases the risk of type I error, and significant findings from subgroup analyses should be interpreted as exploratory. Finally, because operative environments and infection prevention strategies vary substantially across institutions, these findings should not be overgeneralized to specific surgical workflows or interpreted as justification for uniform prophylaxis protocols. Instead, they underscore the need for selective, risk-based application and prospective studies with standardized operative variables.

Nevertheless, this study represents the largest multicenter analysis to date of topical vancomycin use in PSF. Topical vancomycin administration was not associated with a significant reduction in postoperative SSI rates compared with systemic perioperative cefazolin alone in adult patients undergoing instrumented PSF. However, when topical vancomycin was used in combination with topical gentamicin or tobramycin, a lower rate of deep SSI was observed. Further prospective research is warranted to explore the efficacy of dual agent topical antibiotic treatment in infection risk reduction.

## Supplementary Information

Below is the link to the electronic supplementary material.

Supplementary file1 (DOCX 17 KB)

## Data Availability

Not applicable.
