# Supplementary material for: Comparison of postoperative infection risk with and without topical vancomycin in adult posterior spinal fusion: a propensity-matched cohort study of 126,910 patients
Source: Spine Deform. 2026 Apr 27;14(5):1629–37. doi: 10.1007/s43390-026-01378-y (PMC13553626; doi:10.1007/s43390-026-01378-y)
Supplement: Supplementary file 1 [file 43390_2026_1378_MOESM1_ESM.docx]

Supplemental Digital Content 1. Cohort Characteristics Before and After Propensity Score Matching

MRSA: methicillin-resistant Staphylococcus aureus

| Variable | Before Propensity Score Matching | | | After Propensity Score Matching | | |
| --- | --- | --- | --- | --- | --- | --- |
|  | Topical | Control | P Value | Topical | Control | P Value |
| Age at Index Operation | 58.7 ± 18.3 | 57.3 ± 19.2 | < 0.001 | 58.7 ± 18.3 | 58.7 ± 18.3 | 0.972 |
| Male | 33165 (52.3) | 52503 (51.7) | 0.165 | 33165 (52.3) | 33158 (52.3) | 0.969 |
| Female | 30281 (47.7) | 48620 (47.9) | 0.162 | 30281 (47.7) | 30287 (47.7) | 0.973 |
| Hypertension | 34323 (54.1) | 51994 (51.2) | < 0.001 | 34323 (54.1) | 34323 (54.1) | 1.000 |
| Diabetes | 15069 (23.7) | 22092 (21.8) | < 0.001 | 15069 (23.7) | 15074 (23.8) | 0.974 |
| Chronic Kidney Disease | 6589 (10.4) | 9526 (9.4) | < 0.001 | 6589 (10.4) | 6579 (10.4) | 0.927 |
| Ischemic Heart Disease | 11825 (18.6) | 17517 (17.3) | < 0.001 | 11825 (18.6) | 11820 (18.6) | 0.971 |
| Cancer | 19234 (30.3) | 29916 (29.5) | 0.002 | 19234 (30.3) | 19220 (30.3) | 0.932 |
| Past MRSA Infection | 528 (0.8) | 722 (0.7) | 0.006 | 528 (0.8) | 526 (0.8) | 0.951 |
| Smoking/Tobacco Use | 19672 (31.0) | 34729 (34.2) | < 0.001 | 19638 (30.9) | 19645 (31.0) | 0.563 |
| Overweight or Obesity | 17303 (27.3) | 24974 (24.6) | < 0.001 | 17303 (27.3) | 17296 (27.3) | 0.965 |
